# Chitosan Modified Biochar Increases Soybean (*Glycine max* L.) Resistance to Salt-Stress by Augmenting Root Morphology, Antioxidant Defense Mechanisms and the Expression of Stress-Responsive Genes

**DOI:** 10.3390/plants9091173

**Published:** 2020-09-10

**Authors:** Sajid Mehmood, Waqas Ahmed, Muhammad Ikram, Muhammad Imtiaz, Sammina Mahmood, Shuxin Tu, Diyun Chen

**Affiliations:** 1 Guangdong Provincial Key Laboratory for Radionuclides Pollution Control and Resources, School of Environmental Science and Engineering, Guangzhou University, Guangzhou 510006, China; drsajid@gzhu.edu.cn (S.M.); drwaqas@gzhu.edu.cn (W.A.); 2 School of Civil Engineering, Guangzhou University, Guangzhou 510006, China; 3 Statistical Genomics Lab, College of Plant Science and Technology, Huazhong Agricultural University, Wuhan 430070, China; ikramuaf35@outlook.com; 4 Soil and Environmental Biotechnology Division, National Institute for Biotechnology and Genetic Engineering, Faisalabad 38000, Pakistan; m.imtiazpk92@hotmail.com; 5 Department of Botany, Division of Science and Technology, University of Education, Lahore 54000, Pakistan; sammina.mahmood@ue.edu.pk; 6 College of Resources and Environment, Huazhong Agricultural University, Wuhan 430070, China

**Keywords:** chitosan, biochar, sodium, kinetics, antioxidants, salinity tolerance genes

## Abstract

Soybean is an important oilseed crop that provides high-quality protein and vegetable oil. Salinity constitutes a negative abiotic factor that reduces soybean plant growth, production, and quality. The adsorption of Na^+^ by chitosan-modified biochar (CMB) has a significant effect on salinity but the application of CMB is limited in soybean. In the current study, CMB was used for characterization of physiological, biochemical, and molecular responses of soybean under salt stress. Comparison of CMB and unmodified (as-is) biochar (BR) demonstrated a significant difference between them shown by using Fourier transform infrared spectroscopy (FTIR), scan electron microscopy (SEM), Brunauer–Emmett–Teller (BET), elemental analysis and z-potential measurement. Pseudo-first and second-order better suited for the analysis of Na^+^ adsorption kinetics. The salt-stress reduced the soybean plants growth, root architecture characteristics, biomass yield, nutrients acquisition, chlorophyll contents, soluble protein, and sugar contents, while CMB with salt-stress significantly increased the above parameters. Moreover, CMB also reduced the salinity-induced increase in the Na^+^, glycine betaine (GB), proline, hydrogen peroxide (H_2_O_2_), and malondialdehyde (MDA) levels in plants. The antioxidant activity and gene expression levels triggered by salinity but with the application of CMB significantly further boosted the expression profile of four genes (*CAT*, *APX*, *POD* and *SOD*) encoding antioxidant enzyme and two salt-tolerant conferring genes (*GmSALT3* and *CHS*). Overall, these findings demonstrate the crucial role of CMB in minimizing the adverse effects of high salinity on soybean growth and efficiency of the mechanisms enabling plant protection from salinity through a shift of the architecture of the root system and enhancing the antioxidant defense systems and stress-responsive genes for achieving sustainable crop production.

## 1. Introduction

Soybean (*Glycine max* L.) is an important oilseed crop that provides 30% oil and 69% protein in the world wide food production [1]. The major portion of oil and soy meal is consumed as edible oil and in animal feed, respectively, while soybean seeds are used in different food items such as tofu, miso, and natto [2]. Salinity is among the greatest threats hindering global food security [3] and higher levels of salinity have a negative impact on soybean production and quality [4]. Moreover, a high degree of salinity induces oxidative stress and ion imbalance in plant cells and limit further plant growth and development [5]. Further, increased Na^+^ prevents the absorption of other nutrients and contributes to a significant nutrient deficit in the plant tissues [6]. According to [7] the concentrations of high salt substantially decrease levels of K^+^, Ca^2+^, Mg^2+^ and other cations that play a major role in photosynthesis. Excessive Na^+^ directly affects the nutrient absorption by interfering with root plasma membrane transporters and/or by inhibiting the root growth. Thus, the effect of salinity is osmotic, ionic, and nutritionally detrimental to plant growth [8]. Therefore, it is challenging to reduce the salinity effect for the better plant growth and yield as well as for the food requirement of the fast-growing world population.

Long-term management strategies are required to reduce the salinity effect in salt-affected soils [9]. As a sustainable technology, the application of biochar (charcoal made from biomass via pyrolysis and used for soil amendments due to its capacity to absorb, e.g., toxic ions including sodium) has generated a rising interest in improving physicochemical characteristics directly associated to Na^+^ removal, for example, Na^+^ adsorption ratio, Na^+^ leaching, and CEC (Cation Exchange Capacity) [10]. Moreover, biochar could improve the soil biological properties under biotic and abiotic stresses conditions [11]. Therefore, short-term management approaches such as biochar addition are useful options to increase farm income [9].

Chitosan (linear polysaccharide composed D-glucosamine and N-acetyl-D-glucosamine and obtained by treating the chitin shells of shrimp and other crustaceans with an alkaline substance) is the world’s most abundant natural polysaccharide and is used as an affordable, safe, and nontoxic product [12]. Consequently, chitosan is a popular remediation agent in many environmental and industrial applications [13]. Adsorbent like chitosan obtained by deacetylation of chitin, is a nontoxic, biodegradable, and biocompatible natural polymer and can be used in a wide range of applications such as in the areas of biomedicine, membranes, drug delivery systems, hydrogels, water treatment, and food packaging. Hence chitosan can be considered as a perfect adsorbent due to bottomless free amino and hydroxyl bunches on its spine. Chitosan and its derivate forms have potential applications in the fields of biotechnology, biomedicine and cosmetics because of their unique properties such as hydrophilicity, biocompatibility, biodegradability and anti-bacterial property [14]. Similarly, furfural biochar increased the CEC, SOC (soil organic carbon) and available P, and decreased pH in saline conditions [15]. Composted biochar increased the soil organic matter content and CEC and decreased the exchangeable Na^+^ and soil pH when applied in saline soils [16]. The above discussion suggested that biochar addition in saline soils could enhance the plant growth by improving the physicochemical properties. Currently, there is minimal or no work on the adsorption of NaCl by chitosan-modified biochar (CMB) in soybean to our full awareness. Several membrane cation antiporters, mainly Na^+^ transporters, play an essential role in plant salt stress resistance mechanisms. For example, the vacuolar Na^+^/H^+^ antiporter in *Arabidopsis* (*AtNHX1*) functions in pumping Na^+^ from cytoplasm into vacuole. The overexpression of the *AtNHX1* gene leads to increased plant salinity tolerance [17]. In previous studies, several Na^+^/H^+^ antiporter and candidate genes have been reported in soybean associated with salt tolerance, such as *GmNHX1* [18], *GmSOS1* [19], *GmSALT3* [20], *GmCAX1* [21], and *GmPIP* [22]. Plants combat osmotic stress with low molecular weight compatible solutes along with an array of non-enzymatic and enzymatic antioxidants like peroxidase (POD), superoxide dismutase (SOD), ascorbate peroxidase (APX), ascorbic acid (AsA), and catalase (CAT). These antioxidants revealed up or down-regulation under stress conditions and confer tolerance to plants [23]. Stress-induced activity of enzymatic antioxidants was also reported in *Brassica juncea* [23], *Zea mays* [24], *Sesamum indicum* [25], and *Cicer arietinum* [26]. Similarly, metal stress also enhanced the expressions of SOD genes in soybean seedlings [27]. Enhanced *APX, POD* gene expression profile in response to toxic metal stress has been reported in perennial ryegrass [28].

Under environmental stresses such as salinity, the yield of soybean is reduced significantly, especially during the early stage of vegetation growth [29]. Improving plant resistance to salt stress by conventional techniques needs additional efforts that are very time consuming. Consequently, the selection and application of modified biochar are important to improve salt-stress. In this study, we examined how the application of chitosan modified biochar (CMB) mediates a response to salt stress in soybean. The objectives of the present study were (a) to find out whether the application of CMB could improve crop nutrient efficiency through root architecture modifications and plant growth under salt stress, (b) to investigate the effect of CMB on physiological response (protein, chlorophyll, soluble sugars, glycine betaine, proline, antioxidant activities, nitrogen and phosphorus contents), biochemical response (antioxidant mechanism), and molecular response (expression analysis of salt-tolerant genes as well as antioxidant genes) of soybean under salt stress.

## 2. Materials and Methods

Rice straw was air-dried, cut and pyrolyzed in a minimal oxygen atmosphere for 2 h at 450 °C to produce as-is biochar, and was subsequently sieved to a uniform 0.5 mm fraction. Before oven drying, at 80 °C, deionized water (DI) had been used to wash away all impurities from the generated as-is biochar and was named as BR after drying. Further, this as-is biochar was modified by chitosan. Briefly, 3 g of chitosan was dissolved in 180 mL acetic acid to prepare the solution then, 3 g of BR was added into the solution and stirred for 30 min. The homogenous suspension of as-is biochar and chitosan was added into a 900 mL NaOH (0.1 N) solution and left for 12 h [14]. The modified biochar was subsequently removed, washed and oven-dried for 24 h and labeled as CMB.

### 2.1. Characterization of Chitosan Modified Biochar

The surface area was determined by Brunauer–Emmett–Teller (BET), and surface area analyzer (AUTOSORB-IMPCR) was used to determine the specific surface area of CMB. Elemental C, H, and N contents of CMB were analyzed by CHN Elemental Analyzer. The percentage yield of biochar (BR) and CMB was determined as the weight ratio of the final adjusted sample to the initial dried raw product. The samples ash was collected and placed in an electric furnace at 800 °C for 4 h with oxidation under atmospheric pressure.

The pH of samples was measured by a pH meter after adding samples into deionized water at a ratio of 1:20. The surface charge characteristics of BR and CMB were estimated from their zeta potential values in solution at pH range 2 to 10. This technique represents the net charge between the surface plane and the slip plane of a colloidal particle [30]. Zetasizer Nano-ZS was used to measure the zeta potential of the amendments. The compositional variations in the samples were carried out by FT-IR spectroscopy (OPUS 2.0 software) at room temperature. Dried samples were mixed with KBr and then pressed into pellets to obtain visible spectra, followed by the samples scanning from 400 to 4000 cm^−1^ with a resolution of 4 cm^−1^. The obtained spectra were analyzed by OMNIC professional software version 8.3. A scanning electron microscope (SEM) was used to take images of BR and CMB to compare the surface morphology of the samples.

### 2.2. Plant Growth

Soybean seeds (*Glycine max* cv. N2899) were obtained from the Chinese National Center for Soybean Improvement because of N2899 known as a salt-sensitive cultivar. Sterilization of seeds was done for 5 min with sodium hypochlorite (10% v/v), repeatedly washed with H_2_O (distilled), and left to germinate at 25 °C for 12 days with Hoagland nutrient solution on wet filter paper [31] along with chitosan modified biochar (CMB) and salt (NaCl) solutions. The growth conditions were as follows: 28/18 °C day/night temperatures, and a 16/8 h day/night photoperiod. Each treatment, which was carried out in triplicate, was covered and incubated with relative moisture of 80% in a growth chamber. The treatments were carried out as follows: (1) control (with no NaCl, CMB and BR treatments) (T1); (2) BR (3%) alone (T2); (3) CMB (3%) alone (T3); (4) NaCl (40 mM) alone (T4); (5) NaCl (40 mM) + BR (3%) (T5); (6) NaCl (40 mM) + CMB (3%) (T6); (7) NaCl (80 mM) alone (T7); (8) NaCl (80 mM) + BR (3%) (T8); and (9) NaCl (80 mM) + CMB (3%) (T9). The plants had been watered with 0, 40 and 80 mM NaCl, 3 times a week. After 12 days of growth, all the plants were harvested, washed thoroughly with water, and dried at 105 °C for 30 min.

### 2.3. Morphological Characteristics of Root and Shoot

Morphological root characteristics were documented, including root length, fresh root weight, shoot length and fresh shoot weight. The plant shoot and root tissues were washed with distilled H_2_O after harvesting, separated and then oven-dried at 75 °C for 48 h to measure the dry weight (DW).

### 2.4. Phosphorus and Nitrogen Contents Calculation

Phosphorus (P) and nitrogen (N) contents in the oven-dried leaves were measured. The powder (dried) (0.2 g) leaf samples were digested for 4 h in 5 mL H_2_SO_4_ solution at 200 °C. The samples were then added to H_2_O_2_ and held for 1 h in a block digester. The digested sample solutions of H_2_O (diluted) were then filtered and the final volume (40 mL) was prepared. The N content was calculated using the Kjeldahl process according to Bremner and Mulvaney [32]. The P content was analyzed by using a spectrophotometer according to the method described by Murphy and Riley [33].

### 2.5. Measurement of Sodium Ion Concentration

After harvesting and washing with deionized water, the root and leaf of soybean plants were separated. The sodium ion concentration measurement followed the procedure of [34]. Briefly, soybean leaves and roots were ground in an IKA mill (A11 basic, IKA) to a powder with uniform particle size. A 0.1 g subsample of dried soybean powder was digested in nitric-perchloric acid (4:1, *v:v*) using a Milestone Ethos T Microwave Digestion System. The digested samples were transferred to a 25 mL volumetric flask and adjusted to a final volume with deionized water. The acids used were trace metal grade (Fisher Scientific, Pittsburgh, PA, USA), and the water was filtered through a MilliQ system (Millipore, Billerica, MA, USA). The concentrations of Na^+^ were determined by inductively coupled plasma optical emission spectrometry (ICP-OES) (CIROS ICP Model FCE12; Spectro, Kleve, Germany).

### 2.6. Estimation of Total Protein, Soluble Sugar, and Chlorophyll Contents

Fresh leaf samples were ground into a cold mortar and macerated into Tris buffer 100 mM (1 mL, pH 8.0). Afterward, the sample extract was centrifuged at 20,000 g and 5 °C for 15 min. The total protein content in the leaf was estimated using the Bradford standard assay [35]. A method described by Rastall [36] was used to measure the total leaf soluble sugar content based on optical density measured by the spectrophotometer at a wavelength of 485 nm.

The total chlorophyll content of leaf was measured by the method of Lichtenthaler and Wellburn [37]. Briefly, 80% acetone (50 mL) was homogenized in a fresh leaf sample (0.2 g) and the extract was centrifuged for 6 min at 15,000× *g*. The optical density of the supernatant was measured at 663 and 645 nm by using a spectrophotometer.

### 2.7. Estimation of the Content of Proline and Glycine Betaine (GB)

Proline content was calculated in leaf with slight modifications in the process, as described by Bates et al. [38]. The fresh leaf sample (0.5 g) was thoroughly homogenized in sulfosalicylic acid (3 mL) by 5% (w/v) and centrifuged at 9000× *g* for 8 min. The supernatant (500 μL) was then diluted to 1 mL with distilled H_2_O and subsequently whirled into two 2% ninhydrin volumes. The blended solution was heated at 95 °C for 35 min. Followed by cooling, toluene in the mixed solution had similar volumes, and the high aqueous phase was used to record the optical density by spectrophotometer at 520 nm wavelength. Proline contents were calculated using the normal L-proline curve. As stated by [39], glycine betaine (GB) in leaf was measured. In brief, the dry leaf content in hot distilled H_2_O was collected at 70 °C, 2 N HCl (0.25 mL) and potassium triiodide solutions (0.2 mL) were carefully added. The mixture was shaken and then cooled for 1.5 h on an ice bath. Afterward, cold distilled H_2_O (2 mL) and 1,2-dichloromethane (20 mL) was added into this mixture, it was thoroughly mixed until two visible layers had been formed. The organic layer absorption was read by using a spectrophotometer at 365 nm wavelength.

### 2.8. Hydrogen Peroxide (H_2_O_2_) and Lipid Peroxidation (MDA) Measurement

Hydrogen peroxide (H_2_O_2_) content was calculated following some minor improvements in the procedures described by [40]. Concisely, a fresh leaf sample (0.5 g) was thoroughly homogenized with 0.1% trichloroacetic acid (TCA, 5 mL). Then extract was centrifuged for 20 min at 10,000 rpm and the supernatant obtained was collected, then 1 M potassium iodide and 10 mM potassium phosphate buffer were mixed in it. The combined solution was then vortexed carefully, and the optical density was measured at 390 nm. The content of H_2_O_2_ was calculated by means of the standard H_2_O_2_ curve with known concentrations as the μM g^−1^ FW. The method in [41], with slight modifications, was used to estimate malondialdehyde (MDA). Briefly, 0.5 g of a fresh sample of the leaf was homogenized in 0.1% TCA (10 mL). The extract obtained was then centrifuged for 7 min at 14,000× *g*. The supernatant (1.5 mL) was thoroughly mixed with 0.5% thiobarbituric acid (TBA) and 6 mL of 20% TCA, heated up to 95 °C for 30 min and then cooled on an ice bath. The mixture was then centrifuged for 10 min at 10,000× *g*, and the optical supernatant density was measured at 532 and 660 nm by using a spectrophotometer.

### 2.9. Antioxidant Enzyme Assays

The ascorbate peroxidase (APX) enzyme activity was calculated using the [42] test. The optical density was measured as per milligram of protein (EU mg^−1^ protein) at 265 nm by a spectrophotometer.

The superoxide dismutase (SOD) enzyme activities in the leaf were measured by following the nitro blue tetrazolium photoreduction method [43]. The optical density was measured at 540 nm and was expressed as the EU mg^−1^ protein.

The activity of Catalase (CAT) was calculated using the [44] test, and the optical density was measured at 240 nm and was expressed as EU mg^−1^ protein. The peroxidase enzyme activity (POD) was measured using the [45] method. The rate of oxidized guaiacol production was noted at 436 nm and was expressed as EU mg^−1^ protein.

### 2.10. Total RNA Isolation and Analysis of Gene Expression Using Quantitative RT-PCR

For this purpose, we selected 12 salt-related genes, *APX* [46], *CAT* [46], *CHS* [47], *SOD* [46], *GmbZIP62* [48], *GmCAX1* [21], *GmHKT1* [49], *GmNHX1* [18], *GmNHX2* [50], *GmSALT3* [20], *GmSOS1* [51], and *POD* [46] for the molecular analysis. Further, we downloaded the RNA-seq data of 14 soybean tissues from Soybase (https://soybase.org/) for the screening of the above-mentioned genes. These genes are comprised of the antioxidant enzymes-encoding genes and some salinity tolerance genes. Then quantitative real-time PCR (qRT-PCR) was used to measure the expression of above genes in soybean plants treated with chitosan modified biochar (CMB) with salt stress (0, 40, 80 mM NaCl). Using the RNeasy Plant Mini kit (Qiagen), the total RNA was extracted from plant tissues and purity/concentration of RNA were examined spectrophotometrically at 260 and 280 nm. Then, using Qiagen Reverse Transcription kit the first strand of cDNA was synthesized. The qRT-PCR reaction was done as follows; (a) total volume of 20 μL, (b) 3 μL of cDNA (4 ng/μL), (c) 0.2 μL of each primer (10 pm/μL), and (d) 10 μL SYBER Green qPCR master mix (Qiagen). The procedures for PCR were as follows: 95 °C for 5 min; 45 cycles of 95 °C for 15 s and 58 °C for 1 min. The qRT-PCR analysis was repeated independently with three replicates. Gene-specific primers used for gene amplification were listed in Table S1. *Actin* was served as a housekeeping gene, and the relative expression levels were determined using the 2^−ΔΔCt^ method.

### 2.11. Sodium Sorption of Chitosan Modified Biochar

The sorption capability of chitosan modified biochar (CMB) for Na^+^ was measured. The efficiency of Na^+^ sorption was determined by adding 0.2 g of a sample to 25 mL of 0.2 M NaCl. The mixture was shaken for 24 h and the solution was filtered using Whatman 42. The Na^+^ concentration was measured with a Corning 410 flame photometer in the filtered solution. The efficiency of Na^+^ sorption was measured as follows:

(1) $$q_{e}=\left(C_{0}-C_{e}\right)\times \frac{V}{m}$$

where *q_e_* (mg g^−1^) is the amount of Na^+^ sorbed at equilibrium, *C*_0_ and *C_e_* (mg L^−1^) are initial, and equilibrium, the concentration of Na ion (mg L^−1^), *V* (L) is the volume of the solution, and *m* (g) is mass of the sample.

The initial NaCl solution molarity ranged from 0.05 M to 0.3 M for the determination of the sorption isotherm. 0.2 g CMB was applied to 25 mL of 0.2 M NaCl for adsorption kinetic tests, and the mixture was shaken for various periods of time. The solution was then filtered, and Na^+^ concentration was measured in the filtered solution. The quantity of sorbed Na^+^ is defined as follows in Equations (2)–(4).

Pseudo-first order:

(2) $$q_{t}=q_{e}\left(1-e^{-k1t}\right)$$

Pseudo-second order:

(3) $$q_{t}=(q_{e}^{2}k_{2}t)/\left(1+q_{e}k_{2}t\right)$$

Intra-particle diffusion:

(4) $$q_{t}=k_{i}t^{0.5}-c$$

where *q_t_* and *q_e_* (mg g^−1^) are the amounts of Na^+^ sorbed at time *t* and equilibrium, respectively, and *k*_1_, *k*_2_, *k_i_* and *c* are constants.

### 2.12. Statistical Analysis

The aov function in R 3.5.0 (http://www.R-project.org/, v3.5.0) was used for one-way analysis of variance (ANOVA) and Duncan multiple range test of investigated traits. The mean values were represented with ± SD (n = 3). Significant effects of biochar applications were identified using the Duncan multiple-range test value at the probability level of 5%.

## 3. Results

### 3.1. Biochar Characteristics

#### 3.1.1. Biochar pH and Elemental Composition

As presented in Supplementary Table S2, the pH, electrical conductivity (EC), cation exchange capacity (CEC) and surface area of the as-is biochar (BR) increased after modification with chitosan (CMB). In contrast, the C and P contents decreased, while the H and N contents increased (Supplementary Table S2).

#### 3.1.2. Biochar Surface Area, Porosity, Surface Functional Groups, and Surface Charge

The scan electron microscopy (SEM) illustrated that the surface morphology of the as-is biochar significantly changed after the modification with chitosan (Supplementary Figure S1A,B). The SEM picture of CMB was the same as in several other BR studies [31,52,53] and showed a highly porous structure of the surface. The surface of CMB was rougher and some crystalline substances (CaPO_4_, NaCl, KCl, NH_4_MgPO_4_. 6H_2_O, CaCO_3_, CaMg (CO_3_)_2_, TiO_2_, SiO_2_, Al_2_O_3_.SiO_2_.H_2_O, FeS/Fe_2_O_3_/Fe_3_O_4_) coated biochar surface and pore as compared to the as-is biochar. Therefore, these results showed that chitosan not only covered the biochar surface during the modification process but penetrated into the biochar pores to affect the biochar porosity structure and these results were similar to Shi et al. [54].

The Fourier transform infrared spectroscopy (FTIR) spectra of the BR and CMB is shown in Supplementary Figure S1C. The BR spectrum in this study shows that only —COOH (1726 cm^−1^), C—O—C (1020 cm^−1^), C—H aromatic (798 cm^−1^) and SiO_2_ free (470 cm^−1^) were present [55]. This finding was linked to the preparation of high-temperature BR, which would minimize aliphatic groups and functional aliphatic groups [56]. Moreover, the FT-IR spectrum of CMB showed increased number of characteristic peaks compared to the BR spectrum. CMB spectrum showed that the functional groups-OH, frame structures—CH, and C-C, respectively, were present at peak 1625, 2883, 798 (cm^−1^), suggested that chitosan was present on the BR as a monolithic coating and these results had been confirmed by a previous study [55].

The surface charge at different pH was analyzed by using Zeta-potential; as a result, CMB had a zero-potential point at pH 9 (Supplementary Figure S1D). The CMB surface charge was positive below 9 pH, whereas the surface charge was negative above pH 9. In contrast, as-is biochar had the highest negative charge, which indicated the contribution of the mineral fraction. Zhao et al. [30] supported these findings, which reported that mineral components in BR were a part of cations. Likewise, Yuan et al. [57] demonstrated that zeta-potential of nine different crop biochars were within the range of 3–7 pH and these were negatively charged.

Based on the SEM, BET, FTIR and Zeta-potential results, chitosan was successfully coated on the BR surface and thus increased the number of polar functional groups on the BR surface and improved BR polarity and hydrophilicity. Moreover, several low molecular chitosan penetrated the pores of the BR and sacrificed the mesoporous portion to form more micropores.

### 3.2. Sorption Isotherm and Kinetics Study

**Figure 1:**
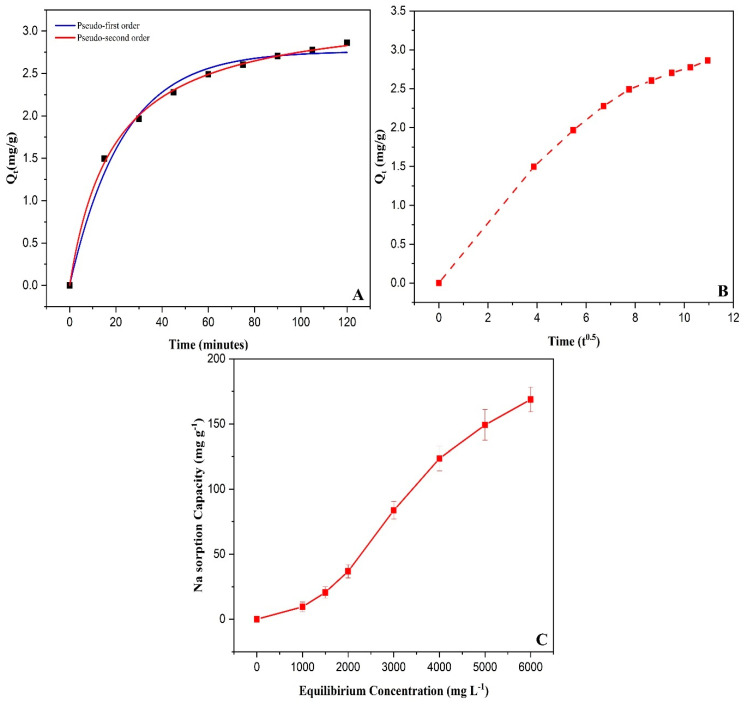
Sorption kinetics of Na^+^ on chitosan modified biochar using (**A**,**B**); sorption isotherm of Na^+^ on chitosan modified biochar (**C**).

Chitosan modified biochar (CMB) was used as a Na^+^ adsorbent, and the amount of Na^+^ adsorbed by CMB increased from 9.49 to 168.77 mg g^−1^, because the initial level of Na^+^ increased from 0 to 6000 mg L^−1^, possibly due to the increased gradient concentration and diffusive movement of Na^+^ to and/or the sorption in higher Na^+^ solutions (Figure 1C).

Na^+^ sorption kinetics on the adsorbent CMB were analyzed by using the pseudo-first-order, pseudo-second-order, and intraparticle diffusion model listed in Supplementary Table S3 and Figure 1A,B. The determination coefficient (R^2^) was used for model evaluation and the values of R^2^ were 0.99, 0.99, and 0.94 for pseudo-first-order, pseudo-second-order, and intraparticle diffusion, respectively. Based on these results, there was a strong relationship between the experimental data and the measured Na^+^ sorbed by pseudo-first and second-order values. These results showed that the intra-particle diffusion of the sorption cycle was less active, suggesting a possible diffusion-controlled process had the lowest agreement with experimental data and was not fitted satisfactorily to the sample.

### 3.3. Plant Growth and Biomass Yield

With the induction of high salinity, soybean plant growth and biomass were substantially reduced and the CMB treatment proved to be beneficial against the effect of salinity (Table 1). In T4 plants (40 mM NaCl) and T7 plants (80 mM NaCl), respectively, root length decreased by 31 and 54%, compared with T1 control plants. However, in comparison of T6 (40 mM NaCl + CMB) and T9 (80 mM NaCl + CMB) with T4 and T7 treatment, respectively, the root length was significantly increased by 56 and 80% (Table 1). While, in comparison of T5 (40 mM NaCl + BR) and T8 (80 mM NaCl + BR) with T4 and T7 treatment, respectively, the root length was significantly increased by BR (by 29 and 31%, correspondingly (Table 1)). In T4 and T7 plants, the fresh root weight was also reduced by 30 and 52%, respectively, compared with control treatment (T1). In T6 plants, the fresh root weight was increased in salt-stressed CMB fertilized soybean by 14%, compared with T4. While the difference between T9 vs. T7 plants were statistically insignificant. However, the addition of as-is biochar in (T5 and T8), insignificantly increased fresh root weight by 9 and 18% compared to T4 and T7, respectively. Dry root weight was decreased by 50 and 70% compared to control (T1) in T4 and T7 plants. Salt treatment with CMB (T6 and T9) and as-is biochar (T5 and T8) significantly increased the dry root weight by 60, 67, 20 and 33% in relation to T4 and T7, respectively. Also, the morphological characteristics of the root were improved by biochar (both modified and as-is) (T2 and T3) relative to T1 under non-stressed conditions (Table 1). Furthermore, salt-stress negatively affected growth and length of the shoot. In T4 and T7 plants, the shoot length was decreased by 24 and 26% compared with the control treatment (T1) (Table 1). Adding CMB to salt-stressed soybean plants in T6 and T9, however, enhanced the shoot length by 38 and 31%, compared with T4 and T7 plants, respectively. Similarly, in comparison of T5 (40 mM NaCl + BR) and T8 (80 mM NaCl + BR) with T4 and T7 plants, respectively, the shoot length was insignificantly increased by 18 and 14%. Comparison of T1 control T4 and T7 plants showed that shoot weights were also decreased by the salt stress. However, the application of CMB and BR with salt-stress in T6 and T9 plants significantly increased shoot weights, in contrast with T4 and T7 plants, respectively. While the difference between T4 vs. T5 and T7 vs. T8 is statistically insignificant. As CMB application with salt-stress significantly increased plant growth and the biomass yield, it is thus highly recommended for the salt-affected soybean plants. Comparison of the effects of CMB with as-is biochar (T6 and T9 vs. T5 and T8, respectively) revealed significantly greater efficiency of modified biochar in terms of longer roots of plants exposed to both concentrations of NaCl. In many cases, the effects of as-is biochar were insignificant, while CMB effects were mainly significant.

### 3.4. Phosphorus and Nitrogen Contents

The use of CMB, under non-stressed conditions (T3), showed a substantial increase in phosphorus and nitrogen content by 59 and 44% respectively, compared to control (T1). Addition of BR under non-stress conditions (T2) did not increase P and N contents significantly. In comparison to T1, high salinity in T7 (80 mM NaCl) plants significantly reduced the absorption of P and N. While the difference between T1 vs. T4 plants were statistically insignificant in terms of P and N contents (Table 1). Comparison of the effects of CMB with as-is biochar in the case of 40 mM and 80 mM NaCl (T6 and T9 vs. T5 and T8, respectively) revealed insignificant efficiency with each other in terms of both P and N contents. While CMB showed a higher percentage of P and N contents than BR as compared to control (T1).

### 3.5. Sodium-Ion Concentration

**Figure 2:**
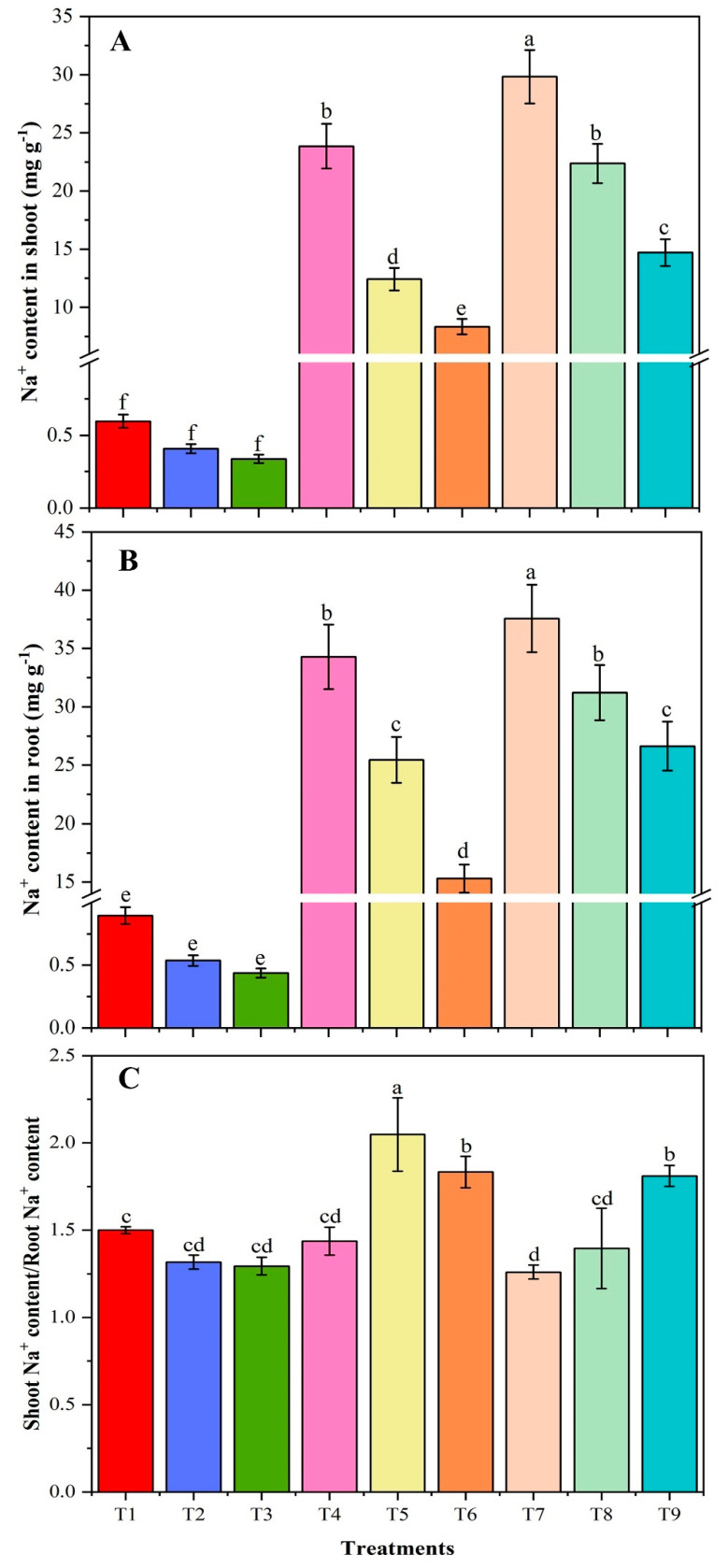
The sodium ion contents in the shoot (**A**) and root (**B**) and shoot/root (**C**) of un-amended and amended soybean plants with chitosan modified and as-is biochar under saline conditions. Data are shown as the mean ± standard deviation (n = 3). Different letters above the chart columns indicate significant differences among treatments (*p* ≤ 0.05). T1, control plants without NaCl, modified and as-is biochar treatments; T2, plants treated with as-is biochar; T3, plants treated with chitosan modified biochar; T4, plants treated with 40 mM NaCl; T5, plants treated with 40 mM NaCl and as-is biochar; T6, plants treated with 40 mM NaCl and chitosan modified biochar; T7, plants treated with 80 mM NaCl; T8, plants treated with 80 mM NaCl and as-is biochar; T9, plants treated with 80 mM NaCl and chitosan modified biochar.

With the induction of high salinity, sodium ion concentration of soybean plant shoot and root were substantially increased and the CMB treatment proved to be beneficial against the effect of salinity (Figure 2). In T4 (40 mM NaCl) and T7 (80 mM NaCl) plants, respectively, root and shoot sodium ion contents increased by 40 and 50 times compared with T1 control plants. However, in comparison of T6 (40 mM NaCl + CMB) and T9 (80 mM NaCl + CMB) with T4 and T7 treatment, respectively, the root sodium content was significantly decreased by 55 and 29%, while shoot sodium content was decreased by 65 and 51% (Figure 2). Similarly, in comparison of T5 (40 mM NaCl + BR) and T8 (80 mM NaCl + BR) with T4 and T7 treatment, respectively, the sodium ion contents in root were significantly decreased by BR (by 26 and 17%), and in the shoot, the sodium contents were decreased by 48 and 25% (Figure 2). The beneficial effect of CMB (extent of the decline in sodium accumulation) was significantly greater than with BR.

### 3.6. Soluble Protein, Soluble Sugar, and Chlorophyll Contents

**Figure 3:**
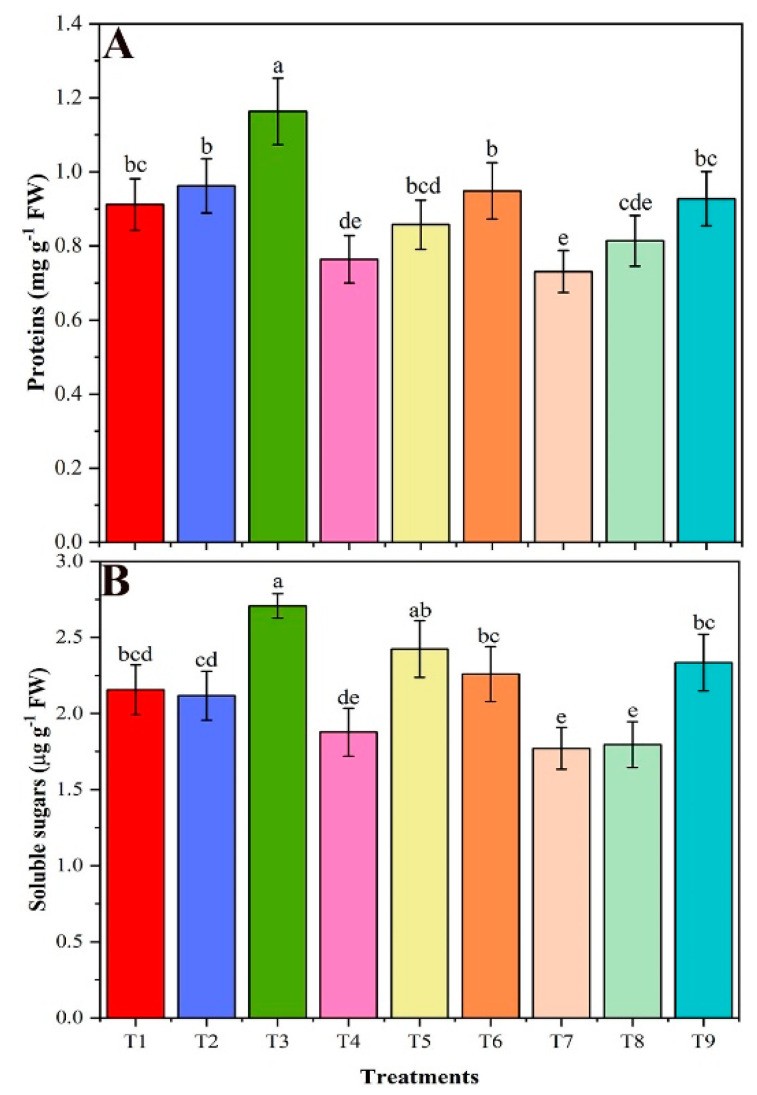
The total protein (**A**) and soluble sugars (**B**) contents of un-amended and amended soybean plants with chitosan modified and as-is biochar under saline conditions. Data are shown as the mean ± standard deviation (n = 3). Different letters above the chart columns indicate significant differences among treatments (*p* ≤ 0.05).

In the present study, the total content of soluble proteins, soluble sugars, and chlorophyll contents was increased by using CMB under salt stress conditions (Figure 3A). During salinity stress, the soluble proteins in T4 (40 mM NaCl) and T7 (80 mM NaCl) were reduced by 16 and 20%, respectively, compared with T1 control (Figure 3A). However, the total content of soluble proteins increased considerably by 24% with the addition of CMB to NaCl-stressed soybean plants at T6 (40 mM NaCl + CMB) and by 27% at T9 (80 mM NaCl + CMB), compared to T4 and T7 treatment, respectively. Effect of BR on soluble protein content was insignificant at both NaCl concentrations. In addition, soluble sugar contents in NaCl-stressed plants (T4 and T7) were respectively 13 and 18% lower than in T1 control (Figure 3B). Application of CMB to salt-stressed plants (T6 and T9 vs. T4 and T7) substantially increased the total soluble sugar content by 20 and 32%, and addition of BR to salt-stressed T5 plants increased total soluble sugar by 29% compared to T4 treatment. Comparison of the effects of CMB with as-is biochar (T6 vs. T5) revealed insignificant efficiency of both the biochars in terms of protein and soluble sugar. While T9 vs. T8 plants revealed significant differences.

**Figure 4:**
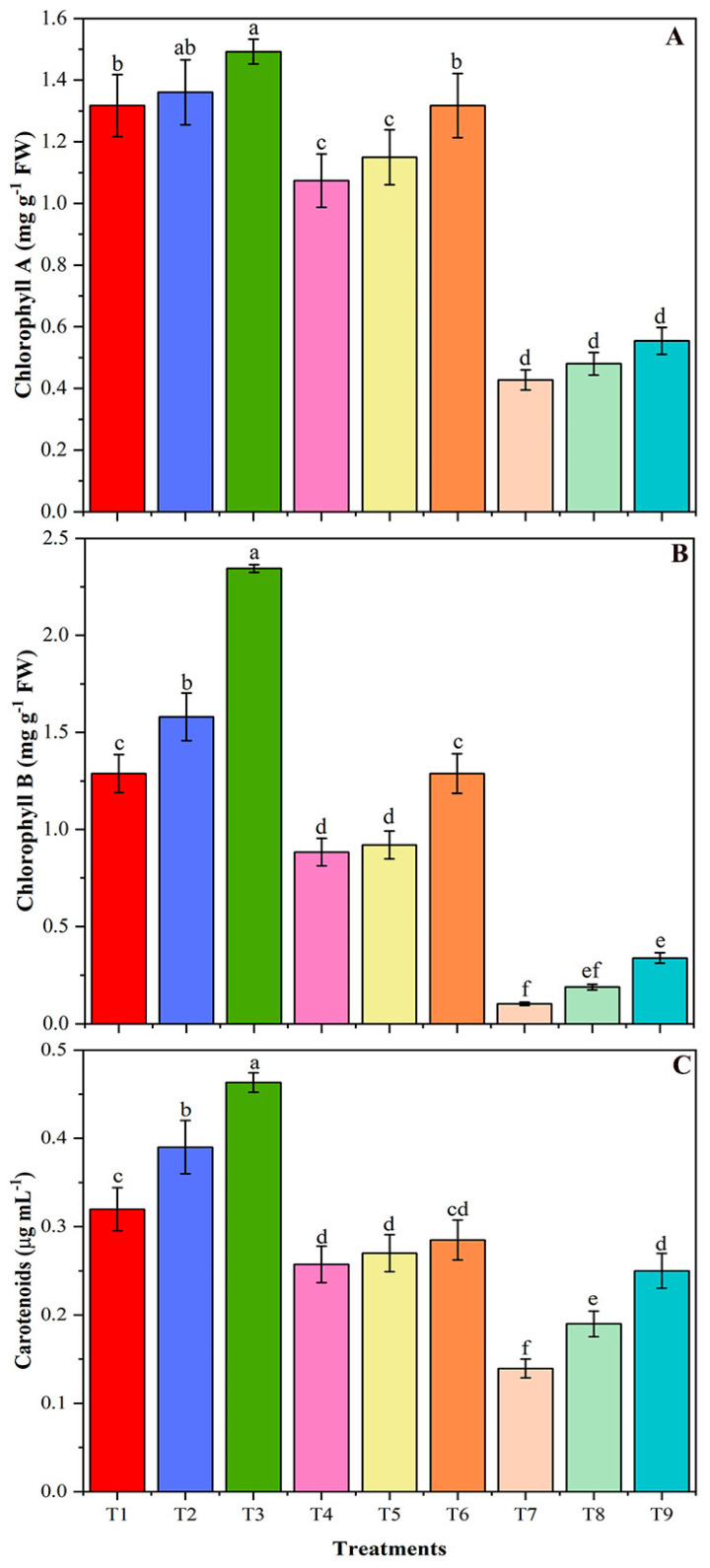
The chlorophyll A (**A**), chlorophyll B (**B**), and carotenoids (**C**) contents of un-amended and amended soybean plants with chitosan modified and as-is biochar under saline conditions. Data are shown as the mean ± standard deviation (n = 3). Different letters above the chart columns indicate significant differences among treatments (*p* ≤ 0.05).

In addition, NaCl-stress reduced the contents of chlorophyll a in T4 and T7, respectively, by 18 and 68% compared with T1 control (Figure 4A). However, application of CMB to NaCl-stressed plants substantially increased chlorophyll a by 23 and 30%, and chlorophyll b by 46 and 230% in T6 and T9, respectively (Figure 4B). The application of BR to salt-stressed soybean plants increased the carotenoids by 36% in T8 plants, compared to T7. While the application of CMB to salt-stressed soybean increased the carotenoids to a greater extent (by 80% in T9 plants, relative to T7 plants (Figure 4C), indicating the greater effect of CMB than BR during salt-stress conditions. Comparison of the effects of CMB with as-is biochar (T6 vs. T5) revealed significantly greater efficiency of the CMB biochar in terms of chlorophyll a and b, while T9 vs. T8 revealed insignificant differences. In terms of carotenoids, T9 vs. T8 plants showed a significant difference, however, the CMB and BR addition to T6 and T5 plants showed the insignificant difference when compared with each other.

### 3.7. Proline and Glycine Betaine Contents

**Figure 5:**
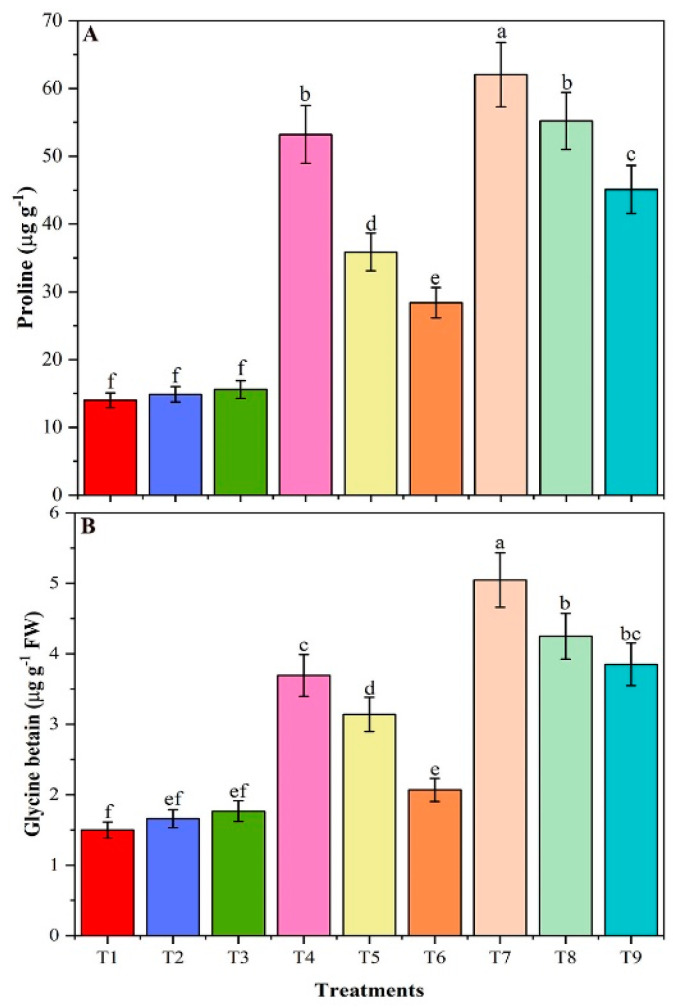
The proline (**A**) and glycine betain (GB) (**B**) and carotenoids (**C**) contents of un-amended and amended soybean plants with chitosan modified and as-is biochar under saline conditions. Data are shown as the mean ± standard deviation (n = 3). Different letters above the chart columns indicate significant differences among treatments (*p* ≤ 0.05).

Application of chitosan modified biochar (CMB) and as-is biochar (BR) decreased the contents of proline and glycine-betaine (GB) under control and NaCl-stress conditions (Figure 5A,B). Salt stress significantly increased the proline content in T4 plants (40 mM NaCl) and T7 (80 mM NaCl) plants, respectively, by 280 and 343% relative to control (T1). At the same time, the proline contents in T6 (40 mM NaCl + CMB) and T9 (80 mM NaCl + CMB) plants significantly decreased by 47 and 27%, respectively, compared with T4 and T7. The application of BR to T5 and T8 plants reduced the amount of proline by 33 and 11% respectively, compared with T4 and T7. In addition, salt stress markedly increased the GB content by 146 and 236% respectively in T4 and T7 plants, compared with T1 control (Figure 5B). The application of BR to T5 and T8 plants reduced the amount of GB by 15 and 16% respectively, compared with T4 and T7, while application of CMB to T6 and T9 plants reduced the amount of GB to a greater extent (by 44 and 24%), respectively. Comparison of the effects of CMB with as-is biochar (T6 and T9 vs. T5 and T8) revealed significantly greater efficiency of the CMB biochar in terms of proline contents. While in terms of GB T5 vs. T6 plants showed significant differences with each other.

### 3.8. Contents of H_2_O_2_ and MDA

**Figure 6:**
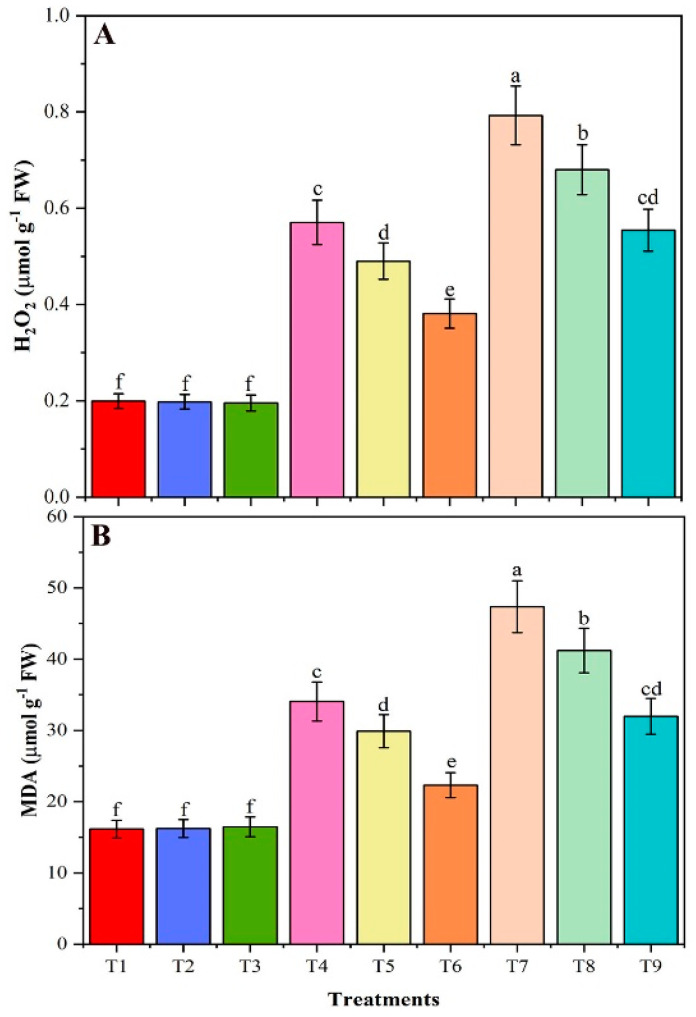
The hydrogen peroxide (H_2_O_2_) content (**A**) and lipid peroxidation (MDA) level (**B**) from leaves of un-amended and amended soybean plants with chitosan modified and as-is biochar under saline conditions. Data are shown as the mean ± standard deviation (n = 3). Different letters above the chart columns indicate significant differences among treatments (*p* ≤ 0.05).

Salt stress substantially increased the H_2_O_2_ content by 186 and 297%, compared with control (T1), in T4 (40 mM NaCl) and T7 (80 mM NaCl) plants, respectively (Figure 6A). However, CMB amendments to NaCl stressed plants significantly decreased the H_2_O_2_ content in T6 plants (40 mM NaCl + CMB) by 33% and T9 plants (80 mM NaCl + CMB) by 30% in comparison with T4 and T7, respectively. Similarly, the application of BR to salt-stress soybean plants decreased the H_2_O_2_ content by 14% in T5 and T8 plants respectively, compared to T4 and T7. In addition, T4 and T7, salt-stressed soybean plants significantly improved the MDA by 111 and 193%, compared with the T1 control, respectively (Figure 6B). Nevertheless, in the T6 and T9 plants, CMB to NaCl-stressed plants alleviated MDA content by 34 and 32% relative to T4 and T7, respectively. Interestingly, alone CMB and BR showed a slight reduction in H_2_O_2_ and MDA content when compared with non-stressed conditions (T1). Comparison of the effects of CMB with as-is biochar (T6 and T9 vs. T5 and T8, respectively) revealed significantly greater efficiency of the CMB biochar in terms of H_2_O_2_ and MDA.

### 3.9. Activities of Antioxidant Enzymes

**Figure 7:**
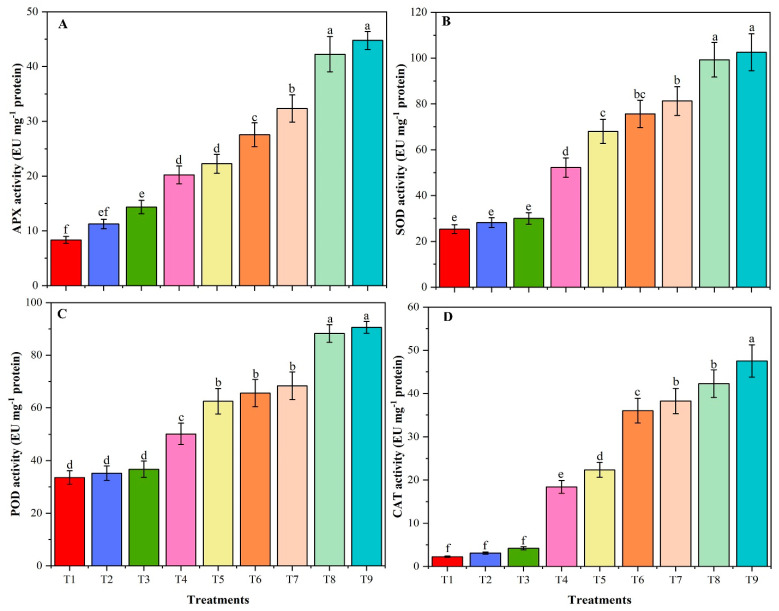
Ascorbate peroxidase (APX) (**A**), superoxide dismutase (SOD) (**B**), peroxidase (POD) (**C**) and catalase (CAT) (**D**) activities of un-amended and amended soybean plants with chitosan modified and as-is biochar under saline conditions. Data are shown as the mean ± standard deviation (n = 3). Different letters above the chart columns indicate significant differences among treatments (*p* ≤ 0.05).

Antioxidants play an essential role in salt-stress by inhibiting the oxidation (chain reactions involving free radicals, thereby leading to chain reactions that may damage the cells of organisms). In the present study, incorporation of chitosan modified biochar (CMB) protected the plants from oxidative stress by increasing activity of antioxidant enzymes (APX, CAT, SOD and POD) under saline stress (Figure 7A–D). Antioxidant enzymes activities were significantly increased in T5, T6, T8 and T9 plants, compared with T4 and T7. Here the results were very interesting, when compared T6 (40 mM NaCl + CMB) with T4 (40 mM NaCl) and T7 (80 mM NaCl), found that antioxidant activities in T6 significantly increased to T4, and non-significantly increased in relation to T7, indicating that CMB significantly enhanced the antioxidant activities under salt conditions. Comparison of the effects of CMB with as-is biochar (T6 vs. T5) revealed significant efficiency of the CMB biochar in terms of APX and CAT activities, while in terms of SOD and POD activities both biochars had insignificant difference. However, T9 vs. T8 revealed insignificant differences in terms of APX, SOD and POD activities, except CAT activity, which showed significant difference.

### 3.10. Screening and Expression Analysis of Antioxidant Enzymes-Encoding Genes and Salt-Related Genes

Using the RNA-seq dataset, eight genes *APX, CAT, SOD, GmbZIP62, GmCAX1, GmNHX1, GmNHX2*, and *GmSOS1*, had two-times higher expression profiles in all 13 soybean plant tissues. Three genes, *POD*, *GmHKT1,* and *GmSALT3* only expressed in root tissue, while *CHS* expressed in five reproductive and vegetative tissues. Further, among eight high expression genes, we selected three genes (*SOD, CAT*, and *APX*) for expression analysis using qRT-PCR because *SOD*, *CAT*, and *APX* genes exhibited a unique higher expression profile in all 13 tissues especially in vegetative tissues such as root, young leaf, flower, pod, and pod shell. Among low expression genes, we selected three genes *POD, GmSALT3* and *CHS* for expression analysis because *POD* and *GmSALT3* genes were rarely or not expressed in all vegetative and reproductive tissues except root, whereas the *CHS* gene was rarely or not expressed in root tissue. Finally, three high expression genes and three low expression genes were preferred for further analysis to confirm the CMB effect. The possible explanation for selection of the above genes is that these genes had different expression profiles in early/vegetative developmental stages, and early developmental stages were significantly disturbed during salinity stress. These stages are more important for plant development/architecture as well as further final yield.

**Figure 8:**
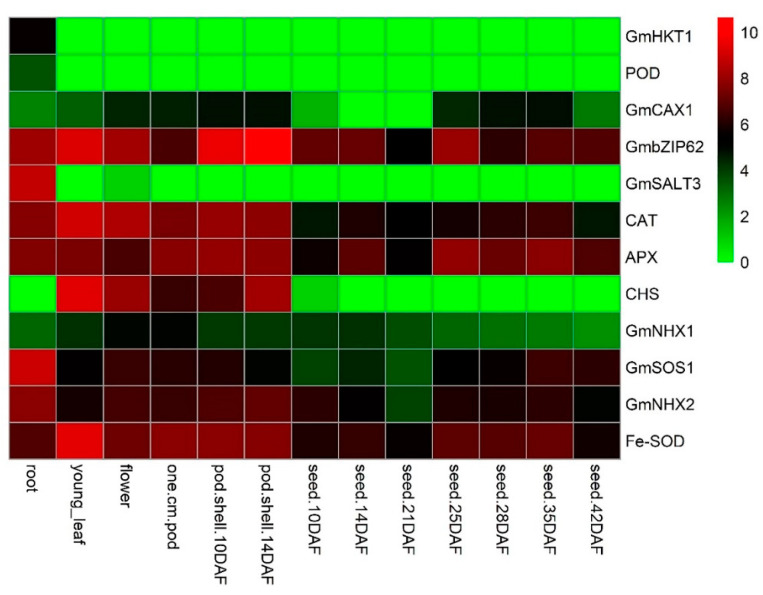
The expressional levels (log2(RPKM + 1)) of antioxidant enzyme-encoding gene and salt-tolerant genes in thirteen soybean tissues.

**Figure 9:**
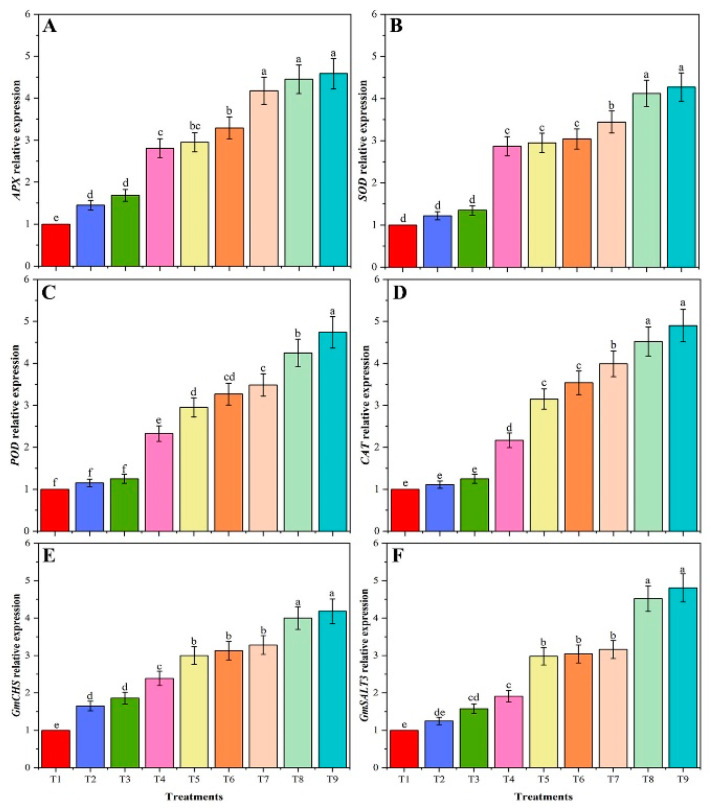
Expression levels of *APX* (**A**), *SOD* (**B**), *POD* (**C**), *CAT* (**D**), *GmCHS* (**E**) and *GmSALT3* (**F**) genes of un-amended and amended soybean plants with chitosan modified and as-is biochar under saline conditions. Data are shown as the mean ± standard deviation (n = 3).

Moreover, the expression of six genes was brought in response to salinity stress in the absence or presence of CMB (Figure 8). The expression level of six genes significantly increased with the application of CMB and BR in salinity stress. The results of the present study were very interesting, for example, application of CMB (T3) alone increased the expression level of all six genes but was statistically non-significant compared to T1. Similarly, the expression level of *SOD, POD, CAT, APX, GmCHS*, and *GmSALT3* genes significantly up-regulated 3.03, 3.48, 3.98, 4.17, 3.18, 3.89-folds, respectively, in soybean plants with T7 treatment, relative to control (T1; Figure 9A–E). Moreover, the expression level of four antioxidant enzyme-encoding genes and two salt-tolerant conferring genes had significantly higher expression levels at T6 and T9 treatment, compared to T4 and T7 treatment, respectively. The expression of all six genes at T6 was non-significant compared to T7 but showed significant expression compared to T1 and T3. Interestingly, the highest expression profile of all six genes except *APX* (antioxidant as well as salt-related genes) were noted for soybean plants exposed to treatment T9 (80 mM NaCl + CMB), compared to plants treated (T7) with salt alone. These results indicated that salinity had a significant influence on the genes.

All antioxidant enzyme-encoding genes showed higher expression profiles in all tissues except *POD*. The salt-related genes also had a higher expression level in tissues except for *GmHKT1* and these genes had a different expression profile in tissues.

## 4. Discussion

Biochar modifications and fortifications are used to improve nutrient retention and crop production, to promote plant growth and environmental stress tolerance by modulating a variety of biochemical, molecular and physiological pathways in plants [58]. The C and P contents decreased, while the H and N contents increased after as-is biochar modification with chitosan (Supplementary Table S2), indicating that chitosan was successfully coated on the biochar, the molar elemental ratios could be used to indicate the aromaticity, polarity, and hydrophilicity of the biochar [59]. The nitrogen-containing functional groups have high polarity and hydrophilicity, which increase the hydrophilicity and polarity of the biochar. Moreover, chitosan itself does not have aromaticity, so the formation of its coating would reduce the overall aromaticity of composite materials, and these results were consistent with Shi et al. [54]. High salinity in the present study alleviates growth, morphological traits, and biomass attributes of soybean, which are in line with the previous studies for *Triticum aestivum* in [60], *Solanum* *Lycopersicum* L. in [61], *Glycine max* L. in [62], *Oryza sativa* in [63], and *Capsicum annuum* in [64]. On the other hand, application of chitosan modified biochar (CMB) to salt-stressed soybean plants significantly enhanced plant growth in comparison to non-amended plants, and these findings are consistent with [65]. For example, the authors of [3] used modified biochar in pepper plants under saline conditions and identified that the modified biochar significantly improved the plant growth and productivity. Similarly, [66] demonstrated that biochar and chitosan enhanced the dry biomass of plants under stress conditions.

In this study, high salinity significantly reduced P and N contents, the addition of CMB to salt-stressed plants had no statistically significant effect on P content, while effects on N were significant only with 40 mM NaCl. The obstruction of Na^+^ accumulation and the improvement of nutrient uptake could be a mechanism of salt stress mitigation in stressed plants supplemented by CMB. Our results are consistent with the study of Abd El-Mageed et al. [3] in pepper plants.

High Na^+^ concentration leads to specific ion toxicity and plant growth inhibition [67]. In this study, the root and shoot Na^+^ contents were substantially increased under salt stress. The beneficial effect of CMB (extent of the decline in sodium accumulation) was significantly greater than with BR. Ning et al. [34] demonstrated that soybean plants require more photosynthetic products in roots under saline stress to absorb sufficient moisture and nutrients to sustain regular growth. Under salt stress conditions, most of the energy acquired by photosynthesis and fixed into carbon compounds are used by plants for general maintenance, whereas only a small proportion (10–40%) is used directly for biomass accumulation [4]. Thereafter, excessive amounts of salt enter the shoot and eventually rise to toxic levels in the older transpiring leaves; this toxicity causes premature senescence, reducing the photosynthetic leaf area of the plant to a level that cannot sustain growth [4]. In contrast, the addition of CMB to salt-stressed plants significantly decreased the contents of Na^+^ in soybean plants root and shoot. The authors of [68] concluded that the application of biochar decreased Na^+^ uptake by lettuce under salt stress. In another study, it decreased Na^+^ uptake in salt-stressed maize [69]. These studies showed that biochar might be effective in reducing Na^+^ uptake by soybean plants grown in saline conditions. Our results indicated significant decreases in chlorophyll a and chlorophyll b under salt stress, which agrees with previous results of [70] on *P. vulgaris* L. of [71] on *Ephedra alata*. The decrease in chlorophyll levels in salt stressed plants has been considered as a typical symptom of oxidative stress [72] and was attributed to the inhibition of chlorophyll synthesis, together with the activation of its degradation by the enzyme chlorophyllase [73]. Reduction of chlorophyll contents, because of either slow synthesis or fast breakdown, indicated that there was a photoprotection mechanism through reducing light absorbance by decreasing chlorophyll contents [74]. Similar findings have been reported in pepper amended with modified biochar [3]. Therefore, the current findings will be useful in future for the improvement of soybean crop under saline conditions.

Plants modulate the mechanisms of self-defense through the accumulation of various osmolytes to reduce the negative impact of salt stress [75]. In the current research, salt stress reduced soluble proteins and soluble sugars, but increased proline and Glycine betaine (GB) to accumulate in the soybean plant. In contrast, the addition of CMB to salt-stressed plants greatly increased the contents of soluble proteins and soluble sugar, while decreased the proline and GB contents. Present study results are similar to previous studies, such as [47] also reported a reduction of soluble proteins under high salinity in soybean. The authors of [76] demonstrated that cell homeostasis was preserved by soluble sugars and changes in soluble sugar content under saline conditions modulate enzyme activity and stress-related gene expression levels. The proline content in the soybean callus dramatically elevated when the NaCl concentration in the medium was increased, suggesting that proline may contribute to the maintenance of low osmotic potential to enhance water uptake [77]. Moreover, in *Linum usitatissimum* [78], *Brassica juncea* L. [79] and *Morus* [80], higher proline and GB contents under salt conditions have also been reported.

In our study, with the increase of salinity levels, the H_2_O_2_ and MDA contents also increased. The enhancement of H_2_O_2_ generation in plants is often observed in response to stress stimuli [81]. Abiotic stress caused overproduction of ROS in the plant cells [82]. A balance between generation and degradation of ROS is required to maintain cellular metabolic functions under stress conditions, thus minimizing oxidative injuries [82]. MDA accumulation serves as an indicator of membrane damage by reactive oxygen species. The CMB application decreased H_2_O_2_ and MDA contents and improved the stability of membranes in plants exposed to NaCl stress. CMB was effective in decreasing oxidative stress due to the improved activity of antioxidant enzymes which efficiently reduced ROS generation and improved plant stress tolerance. Our findings are consistent with the literature that biochar decreased oxidative stress in the plant under stress by activation of antioxidant enzymes [83].

In the present study, antioxidant enzymes, including APX, SOD, CAT and POD were activated by NaCl-stress. Higher activity of antioxidant enzymes caused by salt stress was also observed in *Cicer arietinum* [84]. Similarly, the authors of [82] highlighted that the activity of antioxidant enzymes increased under salinity, compared to non-saline conditions. The use of CMB increased the action of salt-stress induced antioxidant enzymes as well as increased the expression level of four antioxidant genes (*SOD, POD, CAT,* and *APX*) and two salt-tolerant genes (*CHS*, and *GmSALT3)*. The expression level of all six genes was higher with the application of CMB under saline conditions, compared to saline conditions alone, and thereby CMB significantly improved salt tolerance by combating the adverse effect of ROS, accumulating antioxidants, and enhancing the levels of flavonoids and proteins. These findings are in harmony with Ahmed et al. [85], who demonstrated the up-regulation of *SOD, POD, CAT,* and *APX* levels in chickpea under NaCl-stress. The salt-tolerant genes *GmSALT3* and *CHS* had a higher expression profile in the presence of CMB under saline stress and these results are consistent with [47]. Naeem et al. [83] concluded that the amendment of *Chenopodium quinoa Willd.* with modified biochar increased the activities of antioxidant enzymes, and improved stress tolerance. Increasing the antioxidant enzymes may be attributable to soybean plant inhibition of salt adsorption and increased self-adjustment to NaCl toxicity with the application of CMB. Another potential argument may be that the presence of chitosan inhibits ROS and increases immune response through plant expression of the CAT and SOD genes [86]. Therefore, the expression level of these genes during salinity stress and its optimization by CMB suggest that CMB significantly reduce the detrimental effects of salinity by increasing the expression level of antioxidant and salt-related genes thereby enhancing the soybean plant development and stress mechanism. Thus, CMB is suitable for those soils where salinity affects soybean plant growth and yield.

## 5. Conclusions

This study showed that the addition of chitosan modified biochar (CMB) increased salt tolerance in soybean plants. Although the use of high biochar levels may be prohibitive in some regions, the benefits can be higher than the costs in highly saline areas, especially in high-value oil crops. These findings obtained mainly from the improved acquisition of essential nutrients, particularly N and P, which play an important role in osmotic adjustment and cell membrane damage improvement. The plant growth, mineral procurement, biomass yield and chlorophyll content of soybean plants were influenced significantly by salt stress, modulating various molecular and physiological processes. Application of CMB under saline stress has markedly enhanced the soybean growth and the plant performance by osmolyte development, reduced ROS generation, activation of antioxidant protection systems, as well as genetic upregulation that mediates tolerance, including high salinity of abiotic stresses. The integrative application of CMB may thus be a promising and eco-friendly approach to optimizing crop production in salt environments.

## Figures and Tables

**Table 1:** Leaf phosphorus (P) and nitrogen (N) contents and Root and Shoot biomass/growth of un-amended and amended soybean plants with chitosan modified and as-is biochar under saline conditions.

Treatments	P Content	N Content	Root Length	Shoot Length	Root FW	Shoot FW	Root DW	Shoot DW
	%		cm		g			
T1	0.3 ± 0.02 BCD	0.8 ± 0.06 CD	12 ± 0.91 BC	19 ± 1.46 CD	0.9 ± 0.07 B	1.4 ± 0.1 B	0.10 ± 0.01 C	0.27 ± 0.02 B
T2	0.4 ± 0.03 B	0.8 ± 0.06 CD	13 ± 0.99 B	24 ± 1.8 B	0.9 ± 0.07 B	1.5 ± 0.12 B	0.12 ± 0.01 B	0.28 ± 0.02 B
T3	0.5 ± 0.04 A	1.1 ± 0.09 A	20 ± 1.52 A	32 ± 2.45 A	1.4 ± 0.11 A	2.1 ± 0.16 A	0.16 ± 0.01 A	0.42 ± 0.03 A
T4	0.3 ± 0.03 BC	0.7 ± 0.06 D	8 ± 0.69 E	15 ± 1.23 FG	0.6 ± 0.05 C	0.8 ± 0.07 D	0.05 ± 0 E	0.18 ± 0.02 C
T5	0.3 ± 0.03 BC	0.8 ± 0.07 BC	11 ± 0.82 CD	17 ± 1.33 DEF	0.7 ± 0.05 C	1.1 ± 0.08 C	0.06 ± 0 E	0.21 ± 0.02 C
T6	0.4 ± 0.03 B	0.9 ± 0.07 B	13 ± 1.03 B	20 ± 1.62 C	0.7 ± 0.06 C	1.1 ± 0.09 C	0.08 ± 0.01 D	0.20 ± 0.02 C
T7	0.3 ± 0.02 E	0.5 ± 0.04 E	5 ± 0.42 F	14 ± 1.1 G	0.4 ± 0.03 D	0.6 ± 0.05 E	0.03 ± 0 F	0.08 ± 0.01 D
T8	0.2 ± 0.02 DE	0.5 ± 0.04 E	7 ± 0.6 E	16 ± 1.35 EFG	0.5 ± 0.04 D	0.7 ± 0.06 DE	0.04 ± 0 F	0.08 ± 0.01 D
T9	0.33 ± 0.03 E	0.6 ± 0.05 E	9 ± 0.78 D	19 ± 1.46 CDE	0.7 ± 0.05 C	0.9 ± 0.07 D	0.05 ± 0 E	0.10 ± 0.01 D

Data are shown as the mean ± standard deviation (n = 3). Different letters next to numbers in the same column indicate significant differences between treatments (*p* ≤ 0.05). T1, control plants without NaCl and modified biochar treatments; T2, plants treated with as-is biochar; T3, plants treated with chitosan modified biochar; T4, plants treated with 40 mM NaCl; T5, plants treated with 40 mM NaCl and as-is biochar; T6, plants treated with 40 mM NaCl and chitosan modified biochar; T7, plants treated with 80 mM NaCl; T8, plants treated with 80 mM NaCl and as-is biochar; T9, plants treated with 80 mM NaCl and chitosan modified biochar.

## Supplementary Materials

The following are available online at https://www.mdpi.com/2223-7747/9/9/1173/s1, Figure S1: SEM images of (A) as-is biochar; (B) chitosan modified biochar; (C) Fourier Transform Infrared spectroscopy of (FTIR-1) as-is biochar and (FTIR-2) chitosan modified biochar; (D) Zeta potentials of (ZP-1) as-is biochar and (ZP-2) chitosan modified biochar., Table S1: List of primers used for expression profile of antioxidant and salt tolerant genes, Table S2: Basic attributes of as-is biochar and chitosan modified biochar, Table S3: Na adsorption onto chitosan modified biochar using the indicated kinetic models.
