# Supplementary material for: Chitosan Modified Biochar Increases Soybean (Glycine max L.) Resistance to Salt-Stress by Augmenting Root Morphology, Antioxidant Defense Mechanisms and the Expression of Stress-Responsive Genes
Source: Plants (Basel). 2020 Sep 10;9(9):1173. doi: 10.3390/plants9091173 (PMC7569946; doi:10.3390/plants9091173)
Supplement: Supplementary file 1 [file plants-09-01173-s001.pdf]

Supplementary Information

Table S1. List of primers used for expression profile of antioxidant and salt tolerant genes.

Gene Name	Forward and Reverse Primers	Reference
GmSALT3	F: TCCTTGACGCTTGGAGTGTT<br>R: CGGTTGATGAAGGGAAAAC	[33]
APX	F: CGTGACGATGATTGGGAAGT<br>R: TGATAGTGATCTTTCGGACCT	[57]
CAT	F: AGCATCTCACCTGAACTTGAA<br>R: AGGTGAGAGGTTTGTGGCC	[57]
POD	F: TTGAAATAAAC CAAAGGAGTAGT<br>R: AATAATTATTTGAATCTCTTTAAGG	[57]
Fe-SOD	F: ATCTTAGTTATGGTTCTCTTTGT<br>R: ATGGTGTAGAGCCTTTTCATAT	[57]
CHS	F: AGGCTAACAGAGGAGGGTA<br>R: CCAATTTACCGGCTTTCT	[58]
Actin	F: CGGTGGTTCTATCTTGGCATC<br>R: GTCTTTCGCTTCAATAACCCTA	[57]

F and R represent forward and reverse primers, respectively.

Table S2. Basic attributes of as-is biochar and chitosan modified biochar.

Basic Attributes	Unit	BR *	CMB **
pH		8.2	9.1
EC	mS cm^{-1}	5.8	6.3
CEC	cmol (+) kg^{-1}	45	53
Surface area	m^2 g^{-1}	468	167.2
N	%	0.11	1.78
C	%	75.76	61.64
H	%	2.11	4.09
P	%	0.8	0.4
Yield	%	32	24.54
Ash	%	22.8	12.2

* BR= as-is biochar, ** CMB= chitosan modified biochar.

Table S3. Na adsorption onto chitosan modified biochar using the indicated kinetic models.

	Pseudo First-Order			Pseudo Second-Order			Intra-Particle Diffusion		
Chitosan modified biochar	k_1	Q_e	R^2	k_2	Q_e	R^2	k_p	C	R^2
	0.043	2.76	0.99	0.016	3.27	0.99	0.253	0.35	0.94

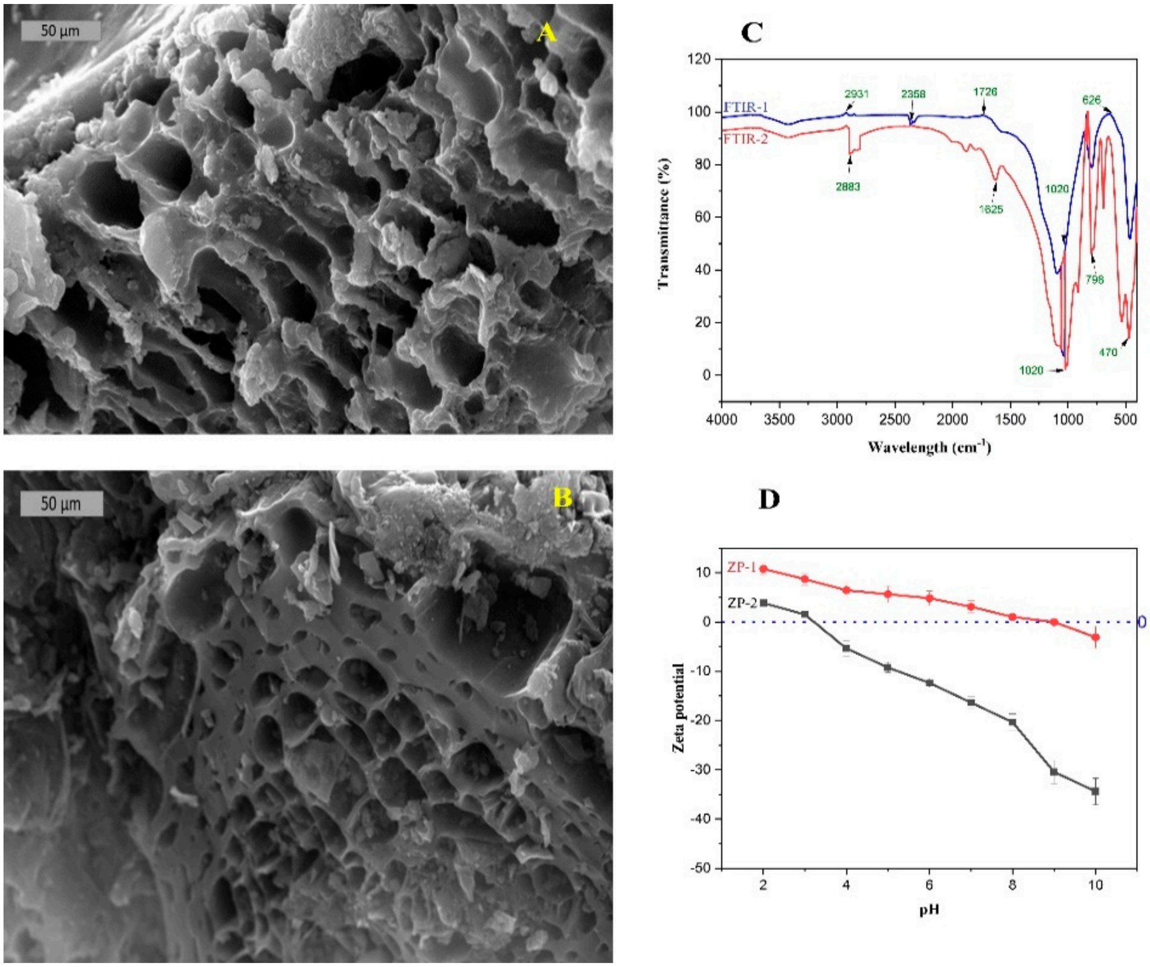

Figure S1. SEM images of (**A**) as-is biochar; (**B**) chitosan modified biochar; (**C**) Fourier Transform Infrared spectroscopy of (FTIR-1) as-is biochar and (FTIR-2) chitosan modified biochar; (**D**) Zeta potentials of (ZP-1) as-is biochar and (ZP-2) chitosan modified biochar.
